# Therapeutic effect of mesenchymal stem cells derived from human umbilical cord in rabbit temporomandibular joint model of osteoarthritis

**DOI:** 10.1038/s41598-019-50435-2

**Published:** 2019-09-25

**Authors:** Hyunjeong Kim, Gwanghyun Yang, Jumi Park, Jene Choi, Eunju Kang, Bu-Kyu Lee

**Affiliations:** 1 Biomedical Engineering Research Center, Asan Institute for Life Sciences, Asan Medical Center, College of Medicine, University of Ulsan, Seoul, Republic of Korea; 2 Department of Stem Cell Center, Asan Institute for Life Sciences, Asan Medical Center, Seoul, Republic of Korea; 3 Department of Pathology, Asan Medical Center, College of Medicine, University of Ulsan, Seoul, Republic of Korea; 4 Department of Stem Cell Center, Asan Institute for Life Science, Asan Medical Center, College of Medicine, University of Ulsan, Seoul, Republic of Korea; 5 Department of Oral and Maxillofacial Surgery, Asan Medical Center, College of Medicine, University of Ulsan, Seoul, Republic of Korea

**Keywords:** Mesenchymal stem cells, Osteoarthritis

## Abstract

Osteoarthritis (OA) is a degenerative condition of the temporomandibular joint (TMJ) characterised by chronic inflammation and damage to joint structures. Because of the complexity of TMJ-OA, only symptomatic treatments are currently available. Recent reports have shown that many of stem cells can exert anti-inflammatory and tissue-regenerating effects. In this study, we investigated the potential cartilage-regenerating and anti-inflammatory effects of human umbilical cord matrix-mesenchymal stem cells (hUCM-MSCs) for the treatment of TMJ-OA. hUCM-MSC lines, isolated from different donors, which showed different activities *in vitro*. Using a selected cell line, we used different concentrations of hUCM-MSCs to assess therapeutic effects in a rabbit model of monosodium iodoacetate-induced TMJ-OA. Compared with the untreated control group, the potential regenerative result and anti-inflammatory effects of hUCM-MSCs were evident at all the tested concentrations in rabbits with induced TMJ-OA. The median dose of hUCM-MSCs showed the prominent cartilage protective effect and further cartilage regeneration potential. This effect occurred via upregulated expression of growth factors, extracellular matrix markers, and anti-inflammatory cytokines, and reduced expression of pro-inflammatory cytokines. The anti-inflammatory effect of hUCM-MSCs was comparable to that of dexamethasone (DEX). However, only hUCM-MSCs showed potential chondrogenesis effects in this study. In conclusion, our results indicate that hUCM-MSCs may be an effective treatment option for the treatment of TMJ-OA.

## Introduction

The temporomandibular joint (TMJ) is a synovial joint involved in complicated movements such as chewing, swallowing, and speaking, which are vital to maintaining life. Osteoarthritis (OA) is a degenerative joint disease characterised by chronic inflammation of the synovial joint and progressive degeneration of the cartilage^1–3^. TMJ is one of the most common sites of OA and is the end stage of TMJ disorders (TMDs). TMJ-OA may cause chronic pain with severe inflammation and discomfort while chewing or speaking, and may even lead to jaw deformity due to joint deformation; this often develops during adolescence compared with OA of other joints. Thus, TMJ-OA can severely reduce the quality of life in affected patients^4^. Approximately 3–7% of the adult population requires treatment for pain and dysfunction stemming from TMJ^5,6^; nearly 11% of individuals with TMDs show symptoms of TMJ-OA^7,8^.

Because of the complex nature of TMJ-OA, the only available therapies are intended for symptomatic management. These include anti-inflammatory drugs, analgesics, soft diets, massaging the jaw muscles, and mouth-opening exercises. Despite these symptomatic approaches, the status of TMJ-OA can worsen, often requiring complicated surgeries such as arthroplasty or total joint replacement surgery, which are stressful for patients both physically and economically^9^. For these reasons, it is necessary to develop an effective therapeutic agent that can regenerate the degenerated cartilage of the joint and reduce inflammation, thereby arresting the progress of TMJ-OA^10^.

Mesenchymal stem cells (MSCs), originating from the bone marrow (BM), adipose tissue, umbilical cord blood, and umbilical cord matrix (UCM or Wharton’s jelly), can differentiate into different cell types. Because of this ability and their immunoregulatory functions, MSCs are currently being tested for the treatment of several clinical conditions including OA^11,12^. Among MSCs, UCMs are more suitable for clinical applications because of their easy obtainability, no donor-site morbidity, young age, abundance in tissues, high expansion potential, low immunogenicity, and high paracrine potential for accelerating tissue- repair processes^13,14^.

In addition to the therapeutic potency of MSCs, the cost and availability of these cells are important factors in broader applications of MSC therapy. Human UCM (hUCM)-MSCs possess several of these intrinsic qualities because they are harvested from discarded tissues, and their cell numbers can be expanded enough to apply to clinical usage without the loss of their stemness properties. Therefore, using hUCM-MSCs for TMJ-OA may be an affordable, safe, and effective therapeutic option. To confirm this hypothesis, we investigated whether hUCM-MSCs can be used for treating TMJ-OA *in vivo* using a rabbit model.

## Results

### hUCM-MSCs analysis with flow cytometry

**Figure 1:**
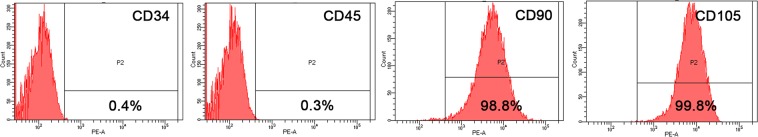
Surface marker analysis by flow cytometry. hUCM-MSCs were negative for CD34 (0.4%) and CD45 (0.3%), and positive for CD90 (98.8%) and CD105 (99.8%).

hUCM-MSCs were phenotyped with flow cytometry. hUCM-MSCs were positive for CD90 (98.8%) and CD105 (99.8%), and negative for CD34 (0.4%) and CD45 (0.3%) (Fig. 1).

### hUCM-MSC selection based on expression of pluripotent markers and capacity to differentiate into chondrocytes

**Figure 2:**
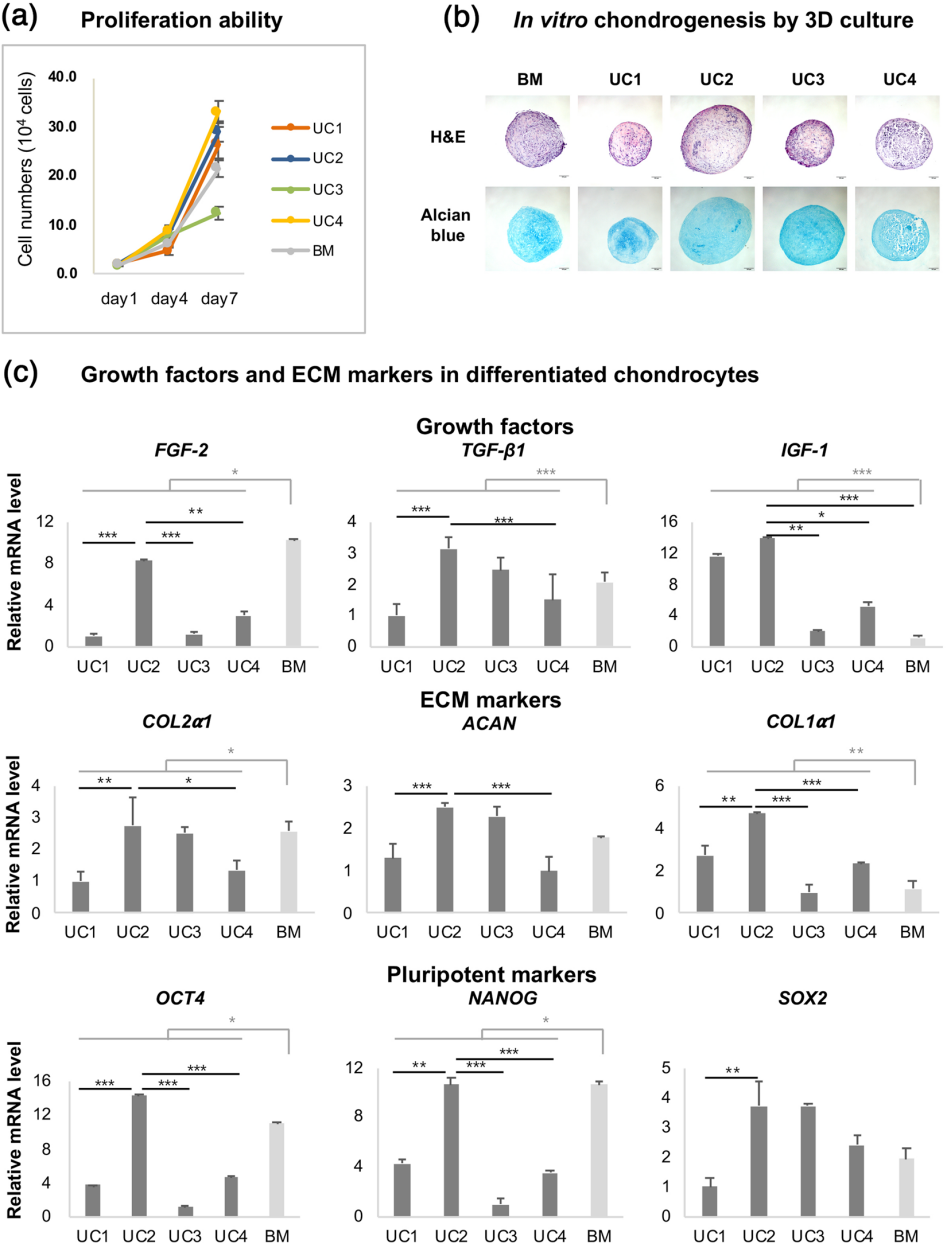
Comparative analysis of therapeutic potency *in vitro*. (**a**) Comparative analysis of proliferation of hUCM-MSCs and hBM-MSCs. The proliferation of hUCM-MSCs was comparable to that of hBM-MSCs, except in the UC3 group. (**b**) Histological images of chondrogenic differentiation in UC and BM pellets. The UC2 and UC3 groups showed marked chondrogenic-differentiation capacity. Scale bar = 100 μm. (**c**) Gene expression related to ECM markers, growth factors, and pluripotency markers in differentiated chondrocytes. The expression levels of *COL2α1* and *NANOG* in the UC2 group were similar to those in the BM group, but higher than those in the other UC groups. The expression levels of TGF- *β1*, *IGF-1*, *ACAN*, *COL1α1*, and *OCT4* in the UC2 group showed the greatest increase in comparison with that of other MSCs, including BM. Biological replicates: *n* = 3; technical replicates: *n* = 2. Data are represented as mean ± SD. ***p < 0.001; **p < 0.01; *p < 0.05. hUCM-MSCs: human umbilical cord matrix-derived mesenchymal stem cells; hBM-MSCs: human bone marrow-derived mesenchymal stem cells; ECM: extracellular matrix; MSCs: mesenchymal stem cells; TGF: transforming growth factor; IGF-1: insulin-like growth factor-1; OCT4: octamer-binding transcription factor 4; SOX2: sex-determining region Y-box 2.

The hUCM-MSC lines were obtained from four individual donors. To assess the therapeutic potential for chondrogenic regeneration *in vitro*, we compared the capacity for proliferation and chondrogenic differentiation in hUCM-MSCs and human BM (hBM)-MSCs, which are well-known standard sources of MSCs. To determine proliferative capacity, the cell numbers of hUCM-MSC and hBM-MSC groups were counted at 1, 4, and 7 days after seeding (Fig. 1). With the exception of cell line UC3, most hUCM-MSCs showed proliferation comparable to that of hBM-MSCs (Fig. 2a).

To determine chondrogenic differentiation capacity, each group of human MSCs (hMSCs) was differentiated into chondrocytes *in vitro*. All hUCM-MSCs were successfully differentiated into chondrocytes after 3 weeks. The UC2 and UC3 pellets demonstrated a marked chondrogenic differentiation capacity, which was characterised by occasional lacunae containing chondrocytes embedded in the extracellular matrix (ECM), and was demonstrated by Alcian blue staining (Fig. 2b).

We used qPCR to compare the expression levels of several genes encoding growth factors, ECM markers, and pluripotent markers of differentiated chondrocytes. Growth factors, such as fibroblast growth factor-2 (FGF-2), transforming growth factor-beta-1 (TGF-β1), and insulin-like growth factor-1 (IGF-1), play key roles in the condensation, proliferation, and differentiation of MSCs in the cartilage^15^. Additionally, the application of TGF-β, IGF-1, and FGF-2 shows promising results as a cell-free approach used to stimulate repair of articular cartilage defects^16,17^. In our study, average *FGF*-2 expression was significantly higher in the BM group than in the UC group (*p* < 0.001); *FGF*-2 expression was comparable between the UC2 and BM groups (Fig. 2c). Conversely, average *TGF*-*β*1 and *IGF*-*1* expression levels were significant higher in the UC group than in the BM group. Furthermore, the UC2 group showed the highest *TGF*-*β1* and *IGF*-1 expression levels among all the UC groups (Fig. 2c).

Chondrogenesis is highly dependent on interactions between cells and the ECM, and on cell–cell adhesions. Thus, the expression of ECM markers, such as collagen type-I alpha-1 chain (*COL1α1*), collagen type-II alpha-1 chain (*COL2α1*), and aggrecan (*ACAN*), can directly influence the regeneration of cartilage^18^. The average *COL1α1* and *COL2α1* expression levels in the UC group were higher than those in the BM group, whereas the expression levels of *ACAN* were comparable between the groups. In particular, the UC2 group showed the highest expression of ECM markers among the UC groups (Fig. 2c).

Pluripotent transcription factors octamer-binding transcription factor 4 (OCT4), NANOG, and sex-determining region Y-box 2 (SOX2), are involved in self-renewal and pluripotency, and are expressed by MSCs^19^. Overexpression of Oct4/Sox2 enhances the anti-inflammatory effects of human adipose tissue-derived MSCs^20^. In agreement with these prior studies, the UC2 group showed the highest *OCT4*, *NANOG*, and *SOX2* expression levels among all the UC groups (Fig. 2c).

### Transplanted hUCM-MSCs alleviate degeneration of cartilage and subchondral bone

**Figure 3:**
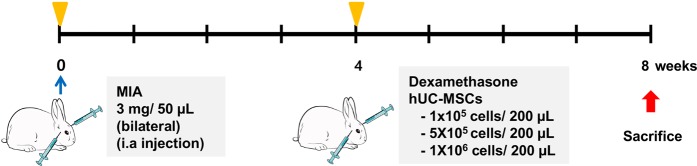
Outline of *in-vivo* study design. Rabbit TMJ-OA was induced by MIA and allowed to stabilize for 1 week. hUCM-MSCs were injected into the TMJs via intra-articular injection at 4 weeks after induction of TMJ-OA. All animals were sacrificed at 8 weeks after induction of OA, and the TMJs were used for radiographical, histological, and molecular analyses. MIA: monosodium iodoacetic acid; TMJ-OA: temporomandibular joint osteoarthritis; hUCM-MSCs: human umbilical cord matrix-derived mesenchymal stem cells; OA: osteoarthritis.

Based on these results, we selected the UC2 group to assess the efficacy of treating TMJ-OA with hUCM-MSCs *in-vivo*. Next, we assessed differences in the regenerative effects of different hUCM-MSC concentrations by comparing three doses of hUCM-MSCs: MSC-L at 1 × 10^5^ cells, MSC-M at 5 × 10^5^ cells, and MSC-H at 1 × 10^6^ cells. All cells doses were suspended in 200 μL saline. The dexamethasone (DEX)-treated group (5 mg/mL, 200 μL), the untreated TMJ-OA induced group (Induced) and the normal condyle group (Control) served as the control (Fig. 3).

**Figure 4:**
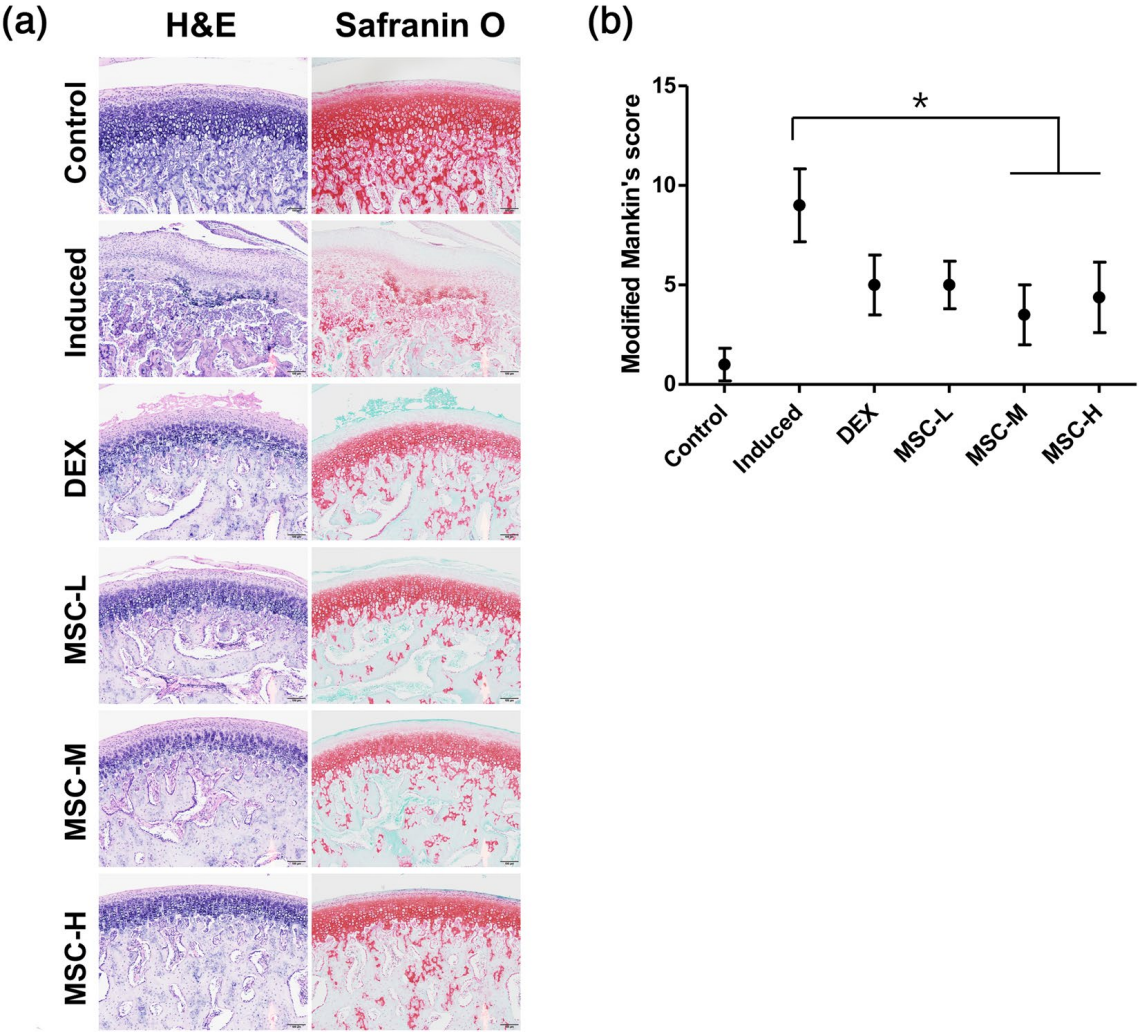
Cartilage regeneration following transplantation of MSCs derived from human umbilical cord. (**a**) Histological images showing fewer surface changes and reduced loss of proteoglycans in the MSC-treated groups compared with those in the untreated TMJ-OA-induced group. The untreated TMJ-OA-induced group demonstrated loss of chondrocytes, cellular disarrangement, abnormal thickening of the fibrous layer, changes in cellularity, disruption of osteochondral junction, necrotic bone debris surrounded by fibrous tissue and osteoclasts, and severe loss of SO staining. The DEX-treated group showed fibrillation of the cartilage surface, whereas MSC-treated groups showed a clear surface and mildly reduced SO staining. Scale bar = 100 μm. (**b**) Modified Mankin scores were significantly higher in the untreated TMJ-OA-induced group than in the control group, but lower in the MSC-treated groups than in the untreated TMJ-OA-induced group. No differences in Mankin scores were noted between the MSC-treated groups. Biological replicates, *n* = 5; technical replicates, *n* = 2. Data are represented as mean ± SD. *p < 0.05. MSCs: mesenchymal stem cells; TMJ-OA: temporomandibular joint osteoarthritis; SO: Safranin O, DEX: dexamethasone.

Figure 4a shows representative photomicrographs of hematoxylin and eosin (H&E)- and Safranin O (SO)-stained sections of articular cartilage acquired from TMJs in each group. The untreated TMJ-OA-induced group (Induced) showed loss of chondrocytes, cellular disarrangement, abnormal thickness of the fibrous layer, changes in cellularity, and disruption of osteochondral junction. We also observed necrotic bone debris surrounded by fibrous tissue and osteoclasts, and a severe loss of SO staining in irregularly arranged chondrocytes. The DEX-treated group (DEX) showed cartilage surface fibrillation, whereas the MSC-treated groups showed clear surfaces and mild reduction in SO staining when compared to the normal condyle(Control) group.

We also evaluated morphological changes in the TMJ articular cartilage based on modified Mankin score^21^ (Table 1). The untreated TMJ-OA-induced group scored significantly higher than the normal control group, but lower than the MSC-M- and MSC-H-treated groups (*p* < 0.05, Fig. 4b). Scores in the DEX- and MSC-treated groups were not significantly different.

**Table 1:** Modified Mankin score.

Parameters	Grade
Pericellular Safranin O staining	
Normal	0
Slightly enhanced	1
Intensely enhanced	2
Background Safranin O staining	
Normal	0
Slight increase or decrease	1
Severe increase or decrease	2
No staining	3
Arrangement of chondrocytes	
Normal	0
Appearance of clustering	1
Hypocellularity	2
Cartilage structure	
Normal	0
Fibrillation in the superficial layer	1
Fibrillation beyond the superficial layer	2
Missing articular cartilage	3

**Figure 5:**
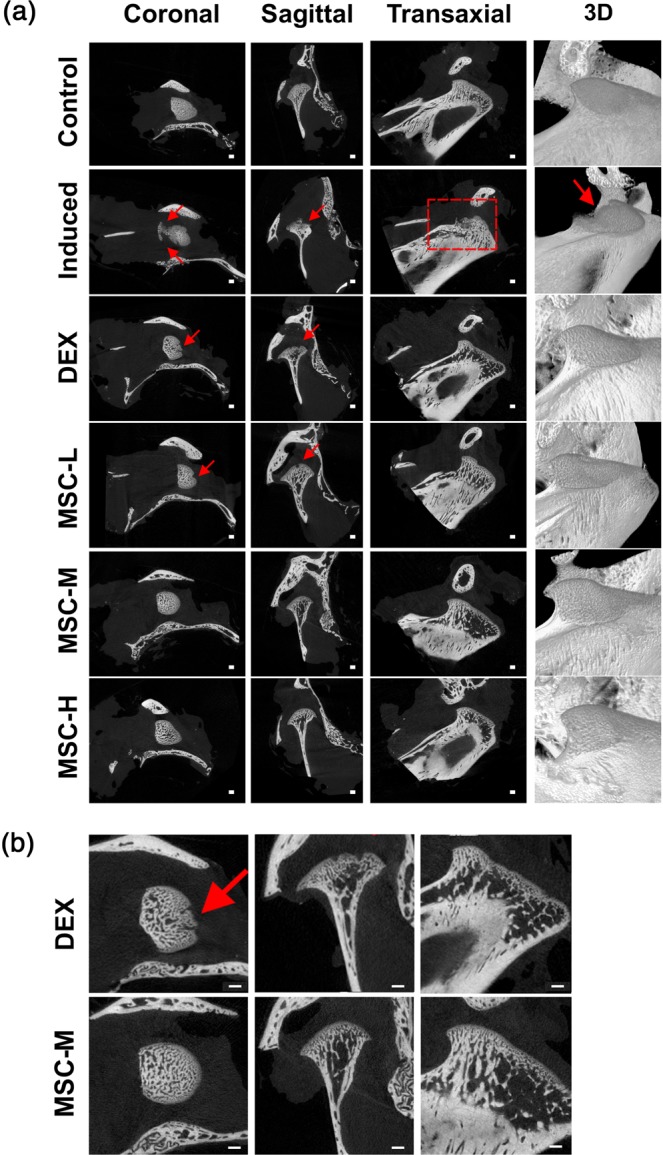
Alleviation of resorption of subchondral bone by hUCM-MSCs. (**a**) Subchondral bone destruction as shown via micro-CT imaging. The surface of the bone in the MSC-treated groups was not significantly different from that in the control group; however, lesions are visible in the untreated TMJ-OA-induced and Dex-treated groups (red arrows and circle). Scale bar = 1 mm. (**b**) Enlarged comparison picture of Dex-treated and MSC-treated groups. micro-CT: micro-computed tomography; DEX: dexamethasone; MSCs: mesenchymal stem cells, RT-PCR: reverse transcription polymerase chain reaction; micro-CT: micro-computed tomography.

Figure 5a illustrates representative images acquired using micro-computed tomography (CT). In both MSC- and DEX-treated groups, losses of condylar bony surfaces were alleviated compared with those in the untreated TMJ-OA-induced group (Fig. 5a). However, the DEX-treated group demonstrated significant regional loss of condylar bony surface, whereas bony surfaces in the MSC-treated groups were smooth and continuous (Fig. 5b)

### hUCM-MSCs suppress OA-induced inflammation as efficiently as does dexamethasone

**Figure 6:**
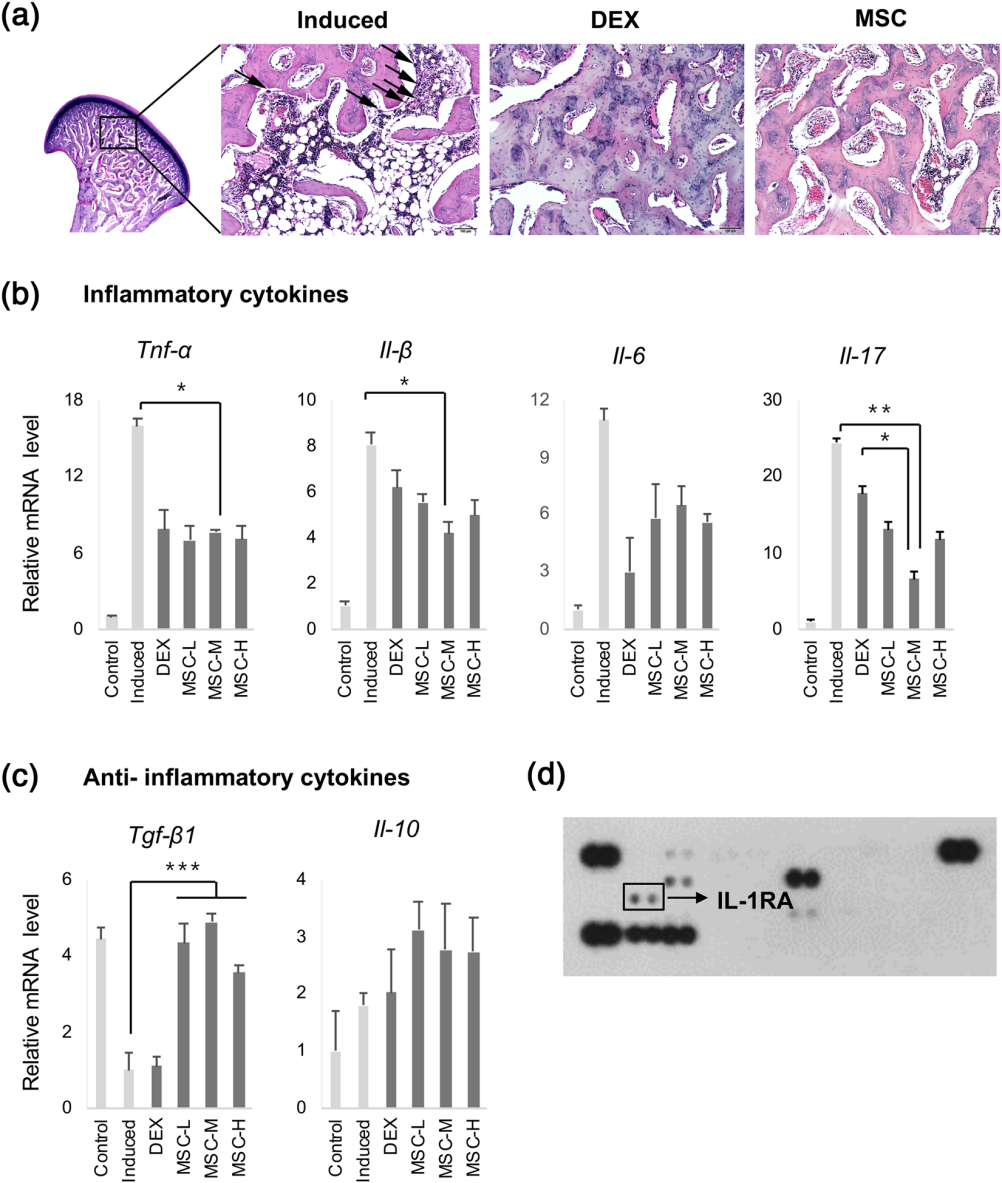
Reduction of inflammation following transplantation of MSCs derived from human umbilical cord. (**a**) Histological images of inflammation in the subchondral bone. The induced group showed resorption of trabeculae and high numbers of vacuoles and inflammatory cells. Lesser inflammation was noted in the MSC-treated groups and in the DEX-treated group than in the induced group. Scale bar = 100 μm. (**b**) The expression levels of genes related to cytokine expression. The expression of pro-inflammatory cytokines *Tnf*-*α*, *Il*-*1β*, *Il*-*6*, and *Il*-*17* was significantly lower in the MSC- and DEX-treated groups than in the induced group. (**c**) Expression of anti-inflammatory cytokines *Tgf*-*β1* and *Il*-*10* was increased in the MSC-treated groups independent of cell concentration. Biological replicates, *n* = 5; technical replicates, *n* = 3. Data are represented as mean ± SD. ***p < 0.001; **p < 0.01; *p < 0.05. (**d**) The secreted inflammation-related cytokines of hUCM-MSCs. IL-1RA were secreted from the selected and injected hUCM-MSCs. TNF: tumour necrosis factor; IL: interleukin; DEX: dexamethasone; MSCs: mesenchymal stem cells; TGF: transforming growth factor; IL-1RA: interleukin-1 receptor antagonist.

Histological observation of subchondral bone inflammation demonstrated both horizontal and vertical resorption of the trabeculae in the untreated TMJ-OA-induced group. We observed high numbers of vacuoles, inflammatory cells, and osteoclasts between the regions of discontinued trabeculae in the untreated TMJ-OA-induced group (Fig. 6a; black arrow). In the MSC-treated group, osteoclasts were also observed in the subchondral bone; however, inflammation was alleviated, and the bony trabeculae in the joint maintained their continuity compared with those in the untreated TMJ-OA-induced group. The morphological appearance of TMJ, observed using H&E staining, was not significantly different between the DEX- and MSC-treated groups (Fig. 6a).

Different inflammatory mediators, such as tumour necrosis factor-alpha (TNF-α), interleukin (IL)-1, and IL-6, are primarily released by stimulated macrophages. These mediators promote bone resorption via osteoclast differentiation and activation, causing acceleration and progression of cartilage degradation. Therefore, these cytokines play crucial roles in the pathogenesis of TMJ-OA^22^. To examine immune-response modulation by hUCM-MSCs, we used qRT-PCR to evaluate the gene-expression levels of these cytokines in the condylar head. The expression levels of pro-inflammatory cytokines *Tnf-α*, *Il-1β*, *Il-6*, and *Il-17* were significantly decreased in the MSC- and DEX-treated groups compared with the levels in the untreated TMJ-OA-induced group (Fig. 6b). Conversely, the expression levels of anti-inflammatory cytokines *Tgf*-*β1* and *Il*-*10* were increased in the MSC-treated groups compared with the levels in the untreated TMJ-OA-induced group (Fig. 6c). To investigate the mechanism of this hUCM-MSC-induced anti-inflammatory effect, we analysed cytokine expression in cell lysates using immunoblotting via human cytokine antibody array. The cytokine array showed that hUCM-MSCs secreted IL-1RA, a well-known natural inhibitor of the pro-inflammatory effects of IL-1*β* (Fig. 6d).

### Upregulation of growth factors and ECM markers by transplanted hUCM-MSCs in chondrocytes of TMJ-OA-induced model

**Figure 7:**
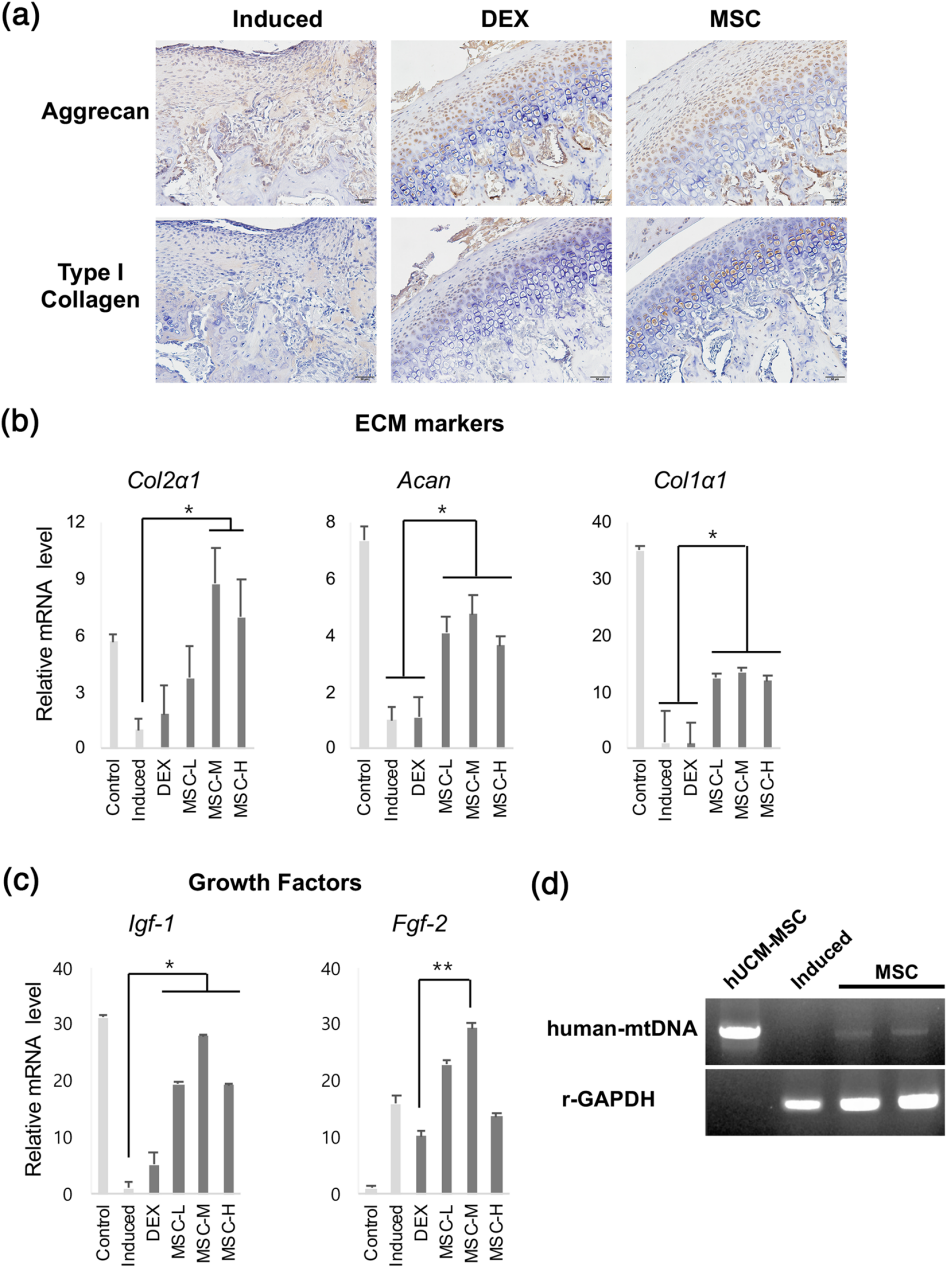
Upregulation of growth factors and ECM markers following transplantation of MSCs derived from human umbilical cord. (**a**) Immunohistochemical labelling for aggrecan and type-I collagen. The expression levels of aggrecan and type-I collagen were greater in the MSC-treated groups than in the untreated TMJ-OA-induced group. Scale bar = 50 μm. (**b**,**c**) The expression levels of genes related to growth factors and ECM markers. The expression levels of *Col2 α1, Acan, Col 1α1, Igf-1 and Fgf-2* were higher in MSC-treated groups, particularly in the MSC-M-treated group (treated at the concentration of 5 × 10^5^ cells/200 µL saline). No significant differences in expression levels were noted between untreated TMJ-OA-induced and DEX-treated groups. Biological replicates, *n* = 5; technical replicates, *n* = 3. Data, mean ± SD. ***p < 0.001; **p < 0.01; *p < 0.05. (**d**) Gel-based RT-PCR gel representing expression of human mtDNA. Human mtDNA was expressed in the hUCM-MSC-injected groups (amplified product size: 1.5 kb). ECM: extracellular matrix; DEX: dexamethasone; IGF-1: insulin-like growth factor-1; FGF-2: fibroblast growth factor-2; MSCs: mesenchymal stem cells; RT-PCR: reverse transcription-polymerase chain reaction; human mtDNA: human mitochondrial DNA.

Representative photomicrographs of IHC are shown in Fig. 7a. The untreated TMJ-OA-induced group showed diffuse positive staining for aggrecan and type-I collagen in chondrocytes adjacent to the lesions. The expression levels of these two factors were greater in the MSC-treated groups than in the untreated TMJ-OA-induced group; no apparent differences in the expression of aggrecan and type-I collagen were noted in the MSC- and DEX-treated groups (Fig. 7a).

The mRNA levels of *Col2α1*, *Acan*, and *Col1α*1 in the condylar heads were significantly upregulated in the MSC-treated groups, particularly in the MSC-M-treated group (5 × 10^5^ cells per 200 μL saline) (*p* < 0.01, Fig. 7b). The expression levels of *Col2α1*, *Acan*, and *Col1α*1 did not differ between the DEX-treated and untreated TMJ-OA-induced groups (Fig. 7b). In addition, the expression levels of these growth factors were significantly higher in the MSC-treated groups than in the untreated TMJ-OA-induced group (Fig. 7c).

We next aimed to confirm that traces of transplanted hUCM-MSCs remained in the lesion. For this, we performed RT-PCR on the condylar heads to determine the expression of human mitochondrial DNA (mtDNA). Our results show that human mtDNA was expressed in the transplanted regions of the hUCM-MSC-treated groups (Fig. 7d).

## Discussion

The treatment approach for TMJ-OA aims to prevent the progressive destruction of cartilage and subchondral bone, relieve joint pain, and restore the function of TMJ. The pathogenesis of TMJ-OA involves a continuous inflammatory process^23^. Metabolic and mechanical factors contribute to a series of biomechanical changes that lead to cartilage injury, causing an inflammatory response in the joint^24^. Thus, treatment for patients with TMJ-OA should focus primarily on suppressing the active inflammatory processes, thereby preventing further degeneration^23^. Secondarily, treatment should address regenerating the damaged cartilage, thereby preserving function. In this study, we investigated the anti-inflammatory effects and cartilage regenerative capacity of hUCM-MSCs in a TMJ-OA rabbit model. In this study, the anti-inflammatory effects of hUCM-MSCs were analogous to treatment with dexamethasone. The therapeutic effects of a single injection of hUCM-MSCs could last for at least 4 weeks until sacrifice. The TMJ condylar cartilage is poorly vascularised; therefore, intraarticular injection rather than intravenous route is the most effective method for delivering MSCs to disease sites. In recent years, administration of MSCs-derived exosome or MSCs supernatant has garnered attention in regenerative medicine because those could avoid numerous hurdles associated with using living cells as therapeutics^25^. However, exosome have been found in multiple studies to possess very short half-lives of 2–5 min in the blood. It is thought that many of these exosomes are quickly digested by macrophages^26^. Thus, therapies using exosome require multiple injections to show therapeutic efficacy^25^. Similar with MSCs derived exosome, MSCs supernatant which contains various cytokines is clinically effective but has similar problems for clinical trials^27^.

In addition, types of bioactive molecules in the MSCs-derived exosome or MSCs supernatant depends on the environment in which the MSCs is located^28^. Therefore, preparation of exosomes or supernatant might be more sophisticated and challengeable to achieve successful clinical results compared with MSCs transplantation.

In this study, we could achieve successful therapeutic effect with single injection of MSCs. This promising result might be due to the fact that transplanted stem cells survive for a considerable period of time and can efficiently home to injured tissue^29^ and communicate with target cells^30^ and release appropriate bio active molecules such as growth factors. Less number of injections is also clinically helpful to reduce patient’s discomfort. As of now, despite of numerous concerns, MSCs therapy has been tested for the treatment in more than 6000 clinical trials and generally found to be safe.

In this study, we examined the therapeutic efficacy of MSCs for the treatment of TMJ-OA. For this, we transplanted different concentrations of hUCM-MSCs into the condylar joint of TMJ-OA-induced New Zealand White male rabbits (2.5–2.8 kg). Our results indicate that MSC-M cells (used at the concentration of 5 × 10^5^ cells per 200 μL saline) were more effective than MSC-H cells (at the concentration of 1 × 10^6^ cells per 200 μL saline) for regenerating articular cartilage. The highest gene-expression level of growth factors was also detected in the MSC-M group (at the concentration of 5 × 10^5^ cells per 200 μL saline). A study performed using rats demonstrated that high-dose treatment with MSCs generated free bodies of scar tissues in the knee^31^. This phenomenon, together with our results, implies that the appropriate number of cells, required to obtain effective treatment, may differ in different OAs; thus, the optimal number of cells for each individual treatment should be established prior to implementing such a treatment.

MSCs, isolated from same tissues of different donors, are heterogeneous and possess different therapeutic effects even if used for the same disease target. In this study, we evaluated multiple hUCM-MSC cell lines from different donors, and conducted a series of *in-vitro* experiments to select an appropriate hUCM-MSC line for the treatment of rabbit TMJ-OA. We assumed that cells with high expression of growth factors would show improved ability to differentiate into chondrocytes via autocrine loop, and would facilitate the regeneration of surrounding cells via paracrine loop. For that reason, we evaluated the chondrogenic differentiation potential of different hUCM-MSC lines. Our results show a positive correlation between the expression levels of growth factors and ECM markers. Growth factors play a role in maintaining the stemness of stem cells^32^. Our data also show that a relationship exists between the expression of growth factors and that of pluripotent markers. In our study, the selected UC2 group showed good performance in the treatment of rabbit TMJ-OA. This result indicates that assessing the *in-vitro* expression of these factors prior to *in-vivo* procedures can help predict the therapeutic effects of MSCs in the treatment of TMJ-OA. Further study is required to clarify the correlation between actual efficacy and expression levels of growth factors and pluripotent markers.

We observed that hUCM-MSC exerted strong anti-inflammatory effects, which were analogous to those of DEX, and occurred via secretion of IL-1RA. However, cartilage regeneration effects were only observed in MSCs-treated groups. ECM markers and growth factors were significantly upregulated in the MSC-treated group. Whereas, the expression of these factors in the DEX-treated group were similar to those in the untreated TMJ-OA-induced group. These results indicate that anti-inflammatory therapy alone has limitations in the treatment of TMJ-OA. In particular, it should be noted that upregulation of mRNA expression of growth factors, especially *Igf-1*, in the hUCM-MSCs group for the following reasons. Unlike hyaline cartilage, which is composed of type II collagen, the articular cartilage of TMJ is fibrous cartilage, composed mainly of type I collagen. Studies have shown that IGF-1 promotes the synthesis of type I collagen^33,34^. Our results show that transplanted hUCM-MSCs promote synthesis of type I collagen via IGF-1 signalling, thereby effectively regenerating TMJ cartilage. This demonstrates that hUCM-MSCs are more suitable for TMJ-OA therapy than for treatment of generic OA.

The cellular fate of transplanted cells remains controversial^35,36^. In this study, we identified human mtDNA of transplanted hUCM-MSCs in samples of rabbit TMJ condylar head 4 weeks after administration of hUCM-MSCs. This suggests that hUCM-MSCs penetrate the cartilage layer and subchondral bone through the degraded ECM, and performed restorative functions for at least 4 weeks. There is also a question whether mesenchymal stem cells promote cartilage regeneration *in vivo* by differentiating into chondrocytes, or by mechanisms that involve the secretion of trophic factors. Daniela *et al*. has shown that MSCs regenerate cartilage not by differentiating into chondrocytes, but via paracrine mechanisms^37^, which can involve immunomodulatory factors, microvesicles, microRNAs, exosomes, and mitochondrial transfer^38^. Based on this fact, our human mtDNA data, acquired using PCR, also an evidence that hUCM-MSCs exerted paracrine effects and restored damaged cartilage by transporting mitochondria or mtDNA to damaged chondrocytes^39^. Mitochondrial dysfunction is related to the pathology of OA^40^. Mitochondrial dysfunction causes decline in cellular activities and accumulation of oxidative stress, leading to degradation of lipid membranes, accumulation of DNA damage, induced catabolic signalling cascades, and chondrocyte apoptosis^41^. Thus, mitochondria transfer is a lucrative method for regeneration of OA, because damaged chondrocytes do not efficiently biosynthesize mitochondria^38^.

Studies report that both autologous and allogenic MSCs are equally safe and therapeutically efficacious in humans^42,43^. Although we used xenogenic stem cells in this study, no local or systemic adverse effects were observed in the experimental animals, indicating that hUCM-MSCs may be safe for future clinical application.

In this study, we induced TMJ-OA in the rabbit model using MIA as previously reported^44^. During TMJ-OA progression, articular chondrocytes with low metabolism usually in advance undergo hypertrophy and apoptosis, accompanied with cartilage fibrillation and progressive loss^45^. Several pro-inflammatory cytokines, such as tumour necrosis factor-alpha (TNF-α), interleukin-1 beta (IL-1β), and IL-6, promote bone resorption via osteoclast differentiation and activation, causing acceleration and progression of cartilage degradation. Therefore, these cytokines play crucial roles in the pathogenesis of TMJ-OA^22^. MIA induce apoptosis of chondrocytes by inhibiting the activity of glyceraldehyde-3-phosphate dehydrogenase, causing subsequent histological changes that are similar to those in human OA^46^.

As observed in the present study, intra-articular MIA injection results in severe inflammation in the TMJ with induction of TNF-α, IL-1β and IL-6. Furthermore, the rabbit model is used to study TMJ-OA because the anatomy and joint movements in rabbits are similar to those in humans^44^. Hence, the MIA-induced TMJ-OA rabbit model is effective and useful in the study of human TMJ-OA.

In conclusion, hUCM-MSCs significantly enhanced cartilage protective effect, further cartilage regeneration potential and exerted anti-inflammatory effects with no local or general side effects in a rabbit model of TMJ-OA. Furthermore, the transplanted cells exert their paracrine effect for a sufficiently long period of time. These results suggest that hUCM-MSCs may be an effective treatment option for the treatment of TMJ-OA.

## Methods

### Ethics statement

This work was approved by the Institutional Review Board of Asan Medical Center (authorization no. 2015–0303) and the Research Ethical Committee of the Geongsang National University Hospital (authorization no. GNUHIRB-2009-34). All participants provided written informed consent. This study was conducted in accordance with the Declaration of Helsinki. All animal experiments were performed in accordance with relevant guidelines and regulations of the Institutional Animal Care and Use Committee of Asan Medical Center (authorization no. 2016-03-099), which is accredited for laboratory animal care by the Ministry of Food and Drug Safety of South Korea.

### hUCM-MSCs and hBM-MSCs

The Institutional Review Board approved the use of these procedures. hUCM-SCs and hBM-MSCs were provided by stem cell laboratories at the Asan Medical Center and Gyeongsang National University. hUCM-MSCs were isolated using a previously described protocol^47^. In brief, human umbilical cord samples without vessels or amnions were minced and digested for 3 h in Minimal Essential Media (MEM; #11095–080; Invitrogen-Gibco, Carlsbad, CA, USA) containing 0.1% collagenase A (#10103578001; Roche, Mannheim, Germany) at 37 °C in a shaking incubator (180 RPM). The cells were filtered through a 70 μM mesh (BD Falcon, San Jose, CA, USA) and pelleted using low-speed centrifugation at 200 *g* for 10 min. Next, the isolated cells were plated in Dulbecco’s modified Eagle’s medium Supplemented with 10% fetal bovine serum, 50 μg/mL penicillin, and 100 μg/mL streptomycin (Invitrogen-Gibco, Carlsbad, CA, USA), and incubated at 37 °C in a humidified incubator at 5% CO_2_.

Isolation of hBM-MSCs was conducted with the approval of the Institutional Review Board of the Gyeongsang National University Hospital (GNUH-2012-09-004). BM aspirates were obtained from the iliac crest using heparin-containing syringes. hBM-MSCs were isolated using a previously described protocol^48^. Briefly, BM aspirates were diluted 1:1 with Dulbecco’s phosphate-buffered saline and layered on Ficoll (GE Healthcare, Freiburg, Germany) at the density of 10.77 g/cm^3^. Mononuclear cells (MNCs) were isolated by density gradient centrifugation at 400 × *g* for 30 min at 22 °C. Isolated MNCs were seeded and cultured on plastic culture plates under MSC maintenance using the same conditions as those used for hUCM-MSCs. hMSCs were selected by adherence to plastic culture plates after 72 h of culture. Non-adherent cells were removed, and growth medium was refreshed every 3 days until 80% confluence was achieved. The hMSC monolayer was detached using trypsin-ethylenediaminetetraacetic acid (EDTA) (0.25% trypsin and 0.53 mM EDTA; Gibco, Carlsbad, CA, USA). All hMSCs undergoing further analysis were used at passages 5–9, and hUCM-MSCs at passages 4 and 5 were pooled for MSC therapy.

### Flow cytometry analysis

Cell membrane protein expression analysis was performed by flow cytometry, using the methods previously described by the present group^48^. hUCM-MSCs were incubated with antibodies for 1 hr in ice-cold blocking buffer. Antibodies against CD34-PE, CD45-PE, CD90-PE and CD105-PE (PE-labeled; BD Biosciences, Franklin Lake, NJ, USA) were used. A total of 25,000 events were acquired with BD FACS CANTO II (Becton Dickinson and Company, Franklin Lakes, NJ, USA).

### Cell proliferation analysis

To assess the proliferative capacity of hMSCs, hMSCs at passage 5 or 6 were seeded and cultured in a 6-well plate at the density of 2 × 10^4^ cells/well under maintenance conditions as described previously. The cells were trypsinised and counted after 1, 4, and 7 days of culture.

### Chondrogenic differentiation

For chondrogenesis, hMSCs were cultured in monolayers according to the manufacturer’s instructions. Briefly, the cells were seeded at a density of 8.3 × 10^3^ cells/cm^2^ in standard growth media. When the cells were 80% confluent, the media in each well were replaced with 2 mL of StemPro Chondrogenesis Differentiation medium (A10071-01; Invitrogen-Gibco, Carlsbad, CA, USA). For the formation of chondrocyte micro-mass, the expansion-sorted primary hMSCs were trypsinised and placed into 24-well plates per manufacturer’s instructions (A10071-01; Invitrogen-Gibco, Carlsbad, CA, USA). Briefly, a 5-µL droplet containing 1 × 10^5^ cells was seeded in the center of each well in a 24-well plate and cultivated. Pre-warmed chondrogenesis media were added to the wells after 2 h of culture, and the media was refreshed every 2–3 days. After 21 days, some of the cells were harvested for RT-PCR, and the remaining cells were stained with Alcian blue to confirm chondrogenesis.

### Histological analysis of 3D culture

Pellets were fixed with 4% paraformaldehyde for 4 h at room temperature, subjected to standard processing and paraffin-embedding, and sectioned at the thickness of 5-µm. Deparaffinized sections were stained with 1% Alcian blue solution in 3% acetic acid at pH 2.5 (Sigma-Aldrich, USA) for 30 min to confirm proteoglycan synthesis.

### Animal procedures and experimental design

All procedures were reviewed and approved by the Institutional Animal Care and Use Committee of the Asan Institute for Life Sciences, Asan Medical Center (No: 2016-03-099) in accord with the guidelines of the Institute of Laboratory Animal Resources. New Zealand white rabbits (2.5–2.8 kg) were provided by Orient Bio Inc. (Gyeonggi, Korea). In total, 25 male rabbits were randomly divided into five groups (Table 1) as follows: (i) NC (not induced control, *n* = 5); (ii) Induced (TMJ-OA induction without treatment, *n* = 5); (iii) DEX-treated (TMJ-OA induction + DEX treatment, *n* = 5); (iv) MSC-L (TMJ-OA induction + hUCM-MSCs at 1 × 10^5^ cells/200 μL saline, *n* = 5); (v) MSC-M (TMJ-OA induction + hUCM-MSCs at 5 × 10^5^ cells/200 μL saline, *n* = 5) and (vi) MSC-H (TMJ-OA induction + hUCM-MSCs at 1 × 10^6^ cells/200 μL saline, *n* = 5). All rabbits were anaesthetised by intramuscular injection of Zoletil (15 mg/kg) and xylazine hydrochloride (3.5 mg/kg). Based on a previously described protocol^40^, TMJ-OA was induced by injecting 3 mg MIA dissolved in 50 µL saline into the upper joint compartment of the bilateral TMJ using a 26-gauge needle. At 4 weeks after induction of TMJ-OA, the groups were administered either 200 μL DEX (Yuhan, Seoul, Korea) or hUCM-MSCs in 200 μL saline via intra-articular injection into the bilateral TMJ. All rabbits were sacrificed at 8 weeks after the induction of TMJ-OA. The entire experimental schedule is illustrated in Fig. 3.

### Animal sacrifice and tissue harvesting

The rabbits were euthanised by injecting 5 mL of KCl into the ear vein. The left condyle was carefully removed and fixed in 10% neutral-buffered formalin. Radiologic images of the fixed samples were acquired by micro-CT. Next, the samples were decalcified in Calci-Clear Rapid (National Diagnostics, Atlanta, GA, USA) and embedded in paraffin for histological evaluation. The right condyle was also removed and immediately frozen in liquid nitrogen prior to qRT-PCR.

### Micro-CT analyses of the subchondral bone

Radiographs of the condyles were obtained using a high-resolution micro-CT system (SkyScan, Kontich, Belgium). TMJ specimens were scanned at 50 kVp (500 µA) using 18-µm isotropic pixel size. Serial reconstructed images were acquired from the raw images using NRecon software (SkyScan). 2D and 3D images were obtained using DataViewer (SkyScan) and CTvox (Bruker) to compare anatomical changes in the articular structure among the groups.

### Histological evaluation

Paraffin-embedded TMJ blocks were sagittally sectioned into 5-µm thick serial sections and stained with H&E and Fast Green/SO. Two observers blindly scored each specimen with reference to the modified Mankin score; this was performed in order to histologically grade the severity of cartilage destruction according to a previously reported protocol (Table 1)^21^.

### Immunohistochemistry

We performed immunohistochemical labelling for aggrecan and type-I collagen. All sections were deparaffinised, rehydrated, subjected to antigen retrieval, and blocked with endogenous peroxidase and 2.5% Normal Horse Serum (Vector Laboratories, Burlingame, CA, USA). Primary antibodies to type-I collagen (mouse monoclonal antibody, 1:200; Abcam) and aggrecan (mouse monoclonal antibody, 1:50; Thermo Fisher Scientific) were applied, and sections were incubated overnight at 4 °C. The samples were then incubated with Horse Anti-Mouse Immunoglobulin G Reagent (Vector Laboratories) at 22 °C. Diaminobenzidine (DAB; Dako Corp., Carpinteria, CA, USA) was used to visualize protein expression on the tissue sections.

### Total mRNA extraction and qRT-PCR

Total RNA from hMSCs, differentiated chondrocytes, and condylar heads was extracted using an RNA Mini Kit (Ambion, Life Technologies, Carlsbad, CA, USA) according to the manufacturer’s instructions. Condylar heads were ground into powder in liquid nitrogen using a mortar and pestle. Reverse transcription was performed using a Superscript VILO cDNA Synthesis Kit (Invitrogen), and qRT-PCR was performed using a Power SYBR Green PCR Master Mix (Applied Biosystems, Foster City, CA, USA) and 7900 real-time PCR System (Applied Biosystems). Amplification specificity was confirmed using melt-curve analysis. PCR was performed using gene-specific primers for TNF-α, IL-1β, IL-6, IL-17, IL-10, TGF-β1, IGF-1, FGF-2, Col2α1, ACAN, Col1α1, and housekeeping gene primer for glyceraldehyde 3-phosphate dehydrogenase (Table 2).

**Table 2:** Primers used for PCR amplicons.

Gene	Forward	Reverse
Human primers		
*COL2A1*	5′-GGCAATAGCAGGTTCACGTACA-3′	5′-CGATAACAGTCTTGCCCCACTT-3′
*ACAN*	5′-GAGGCCAGCAGAGAAGATTCT-3′	5′-GACGCCTCGCCTTCTTGAA-3′
*COL1A1*	5′-TCAGAGAGGAGAGAGAGGCT-3′	5′-ATTCAGGGGAACCTTCGGCA-3′
*FGF-2*	5′-GCGACCCTCACATCAAGC-3′-	5′-AGCCAGTAATCTTCCATCTTCC-3′
*TGF-β1*	5′GCCATTTAATGGCAATGGTAGTCT-3′-	5′-CACAGGGAGCTTGCAGAGAT-3′
*IGF-1*	5′-GATGTATTGCGCACCCCTCA-3′	5′-TTCTGTTCCCCTCCTGGATGT-3′
*GAPDH*	5′-GCCTCAAGATCATCAGCAATGC-3′	5′-TGGTCATGAGTCCTTCCACGAT-3′
*mtDNA*	5′-CCCAAGACAACCAACCAAAA-3′	5′-ACTAGCTTATATGCTTGGGG-3′
Rabbit primers		
*TNF-α*	5′-AGCGCCATGAGCACTGAGA-3′	5′-GCCACGAGCAGGAAAGAGAA-3′
*IL-1β*	5′-TTGAAGAACCCGTCCTCTG-3′	5′-CTCATACGTGCCAGACAACACC-3′
*IL-6*	5′-CTACCGTCCCCACTTCAG-3′	5′-TCCTCAGCTCCTTGATGGTCTC-3′
*IL-17*	5′-TCCCATCCAGCAAGAGTTCCT-3′	5′-AGCCAACGGTCACCCACAC-3′
*IL-10*	5′-GAGAACCACAGTCCAGCCAT-3′	5′-CATGGCTTTGTAGACGCCTT-3′
*TGF-β1*	5′-AAAGGGCTACCACGCCAACCTT-3′	5′-CCGGGTTGTGCTGGTTGTAC-3′
*IGF-1*	5′-AGCTGGTGGATGCTCTTCAGTT-3′	5′-GAAGCAGCACTCATCCACGAT-3′
*FGF-2*	5′-AGCCGAGAGCATCACCAC-3′	5′-GTGGGTCGCTCTTCTCCC-3′
*Col2α1*	5′-AGAAGAACTGGTGGAGCAGCAAGA-3′	5′-TGCTGTCTCCATAGCTGAAGTGGA-3′
Aggrecan	5′-ACACCAACGAGACCTATGACGTGT-3′	5′-ACTTCTCTGGCGACGTTGCGTAAA-3′
*Col1α1*	5′-CTCCGGCTCCTGCTCCTCTAG-3′	5′-GTCCCTCGACTCCTGTGGTTTCC-3′
*GAPDH*	5′-TCACCATCTTCCAGGAGCGA-3′	5′-CACAATGCCGAAGTGGTCGT-3′

### Cytokine antibody array

hUCM-MSCs were washed in cold phosphate-buffered saline (PBS) and resuspended in 5 times the packed cell volume of PBS. Freezing and thawing was repeated three times. Normalised protein content was analysed with a Human Cytokine Array (ARY005B; R&D Systems) according to the manufacturer’s instructions.

### Statistical analysis

Statistical analyses were performed using SPSS Statistics for Windows, version 11.0 (SPSS Inc., Chicago, IL, USA). All data are expressed as mean ± standard deviation. After confirming that the data were normally distributed, all data between the induced and treated groups were analysed using one-way analysis of variance (ANOVA); *p* < 0.05 was considered statistically significant.

## Supplementary information

Supplementary information data

## Data Availability

All data generated or analysed during this study are included in this published article.

## References

[1] Zarb GA, Carlsson GE (1999). Temporomandibular disorders: osteoarthritis. J Orofac Pain.

[2] Doherty M (2001). Risk factors for progression of knee osteoarthritis. Lancet.

[3] Park JH, Jo E, Cho H, Kim HJ (2017). Temporomandibular joint reconstruction with alloplastic prosthesis: the outcomes of four cases. Maxillofac Plast Reconstr Surg..

[4] Liu XW (2011). Insulin-like growth factor-1 suspended in hyaluronan improves cartilage and subchondral cancellous bone repair in osteoarthritis of temporomandibular joint. Int J Oral Maxillofac Surg..

[5] Schiffman EL, Fricton JR, Haley DP, Shapiro BL (1990). The prevalence and treatment needs of subjects with temporomandibular disorders. J Am Dent Assoc..

[6] Carlsson GE (1999). Epidmiology and treatment need for temporomandibular disorders. J Orofac Pain..

[7] Mejersjӧ C, Hollender L (1984). Radiography of the temporomandibular joint in female patients with TMJ pain or dysfunction. Acta Radiol Diagn (Stockh)..

[8] Kim BC, Lee YC, Cha HS, Lee SH (2016). Characteristics of temporomandibular joint structures after mandibular condyle fractures revealed by magnetic resonance imaging. Maxillofac Plast Reconstr Surg..

[9] Tanaka E, Detamore MS, Mercuri LG (2008). Degenerative disorders of the temporomandibular joint: etiology, diagnosis, and treatment. J Dent Res..

[10] Liu L, Li Y (2017). Chondroprotective and anti-nociceptive effects of caffeoylquinic acid in osteoarthritis by downregulating catabolic activity and oxidative damage in chondrocytes. Biomed Pharmacother..

[11] Jo CH (2014). Intra-Articular Injection of Mesenchymal Stem Cells for the Treatment of Osteoarthritis of the Knee: A Proof-of-Concept Clinical Trial. Stem cells..

[12] Bartolucci J (2017). Safety and Efficacy of the Intravenous Infusion of Umbilical Cord Mesenchymal Stem Cells in Patients With Hearth Failure: A Phase 1/2 [Randomized Controlled Trial (RIMECARD Trial Infusion Umbilical Cord Mesenchymal Stem Cells on Cardiopathy)]. Circ Res..

[13] Weiss ML (2008). Immune properties of human umbilical cord wharton’s jelly-derived cells. Stem Cells..

[14] Troyer DL, Weiss ML (2008). Concise review: Wharton’s jelly-derived cells are a primitive stromal cell population. Stem Cells..

[15] Fortier LA, Barker JU, Strauss EJ, McCarrel TM, Cole BJ (2011). The role of growth factors in cartilage repair. Clin Orthop Relat Res..

[16] Lam J, Lu S, Kasper FK, Mikos AG (2015). Strategies for controlled delivery of biologics for cartilage repair. Adv Drug Devil Rev..

[17] Jenniskens YM (2006). Biochemical and functional modulation of the cartilage collagen network by IGF1, TGFbeta2 and FGF2. Osteoarthritis Cartilage..

[18] Mariani E, Pulsatelli L, Facchini A (2014). Signaling pathways in cartilage repair. Int J Mol Sci..

[19] Tsai CC, Hung SC (2012). Functional roles of pluripotency transcription factors in mesenchymal stem cells. Cell cycle..

[20] Li Q (2017). Anti-inflammatory effect of Oct4/Sox2-overexpressing human adipose tissue-derived mesenchymal stem cells. in vivo..

[21] Shen P (2015). Injecting vascular endothelial growth factor into the temporomandibular joint induces osteoarthritis in mice. Sci Rep..

[22] Wang. XD, Zhang JN, Gan YH, Zhou YH (2015). Current understanding of pathogenesis and treatment of TMJ osteoarthritis. J Dent Res..

[23] Egloff C, Hügle T, Valderrabano V (2012). Biomechanics and pathomechanisms of osteoarthritis. Swiss Med Wkly..

[24] Wang XD, Kou XX, Mao JJ, Gan YH, Zhou YH (2012). Sustained inflammation induces degeneration of the temporomandibular joint. J Dent Res..

[25] Zhang. S (2016). Exosomes derived from human embryonic mesenchymal stem cells promote osteochondral regeneration. Osteoarthritis Cartilage..

[26] Willekens (2005). Liver Kupffer cells rapidly remove red blood cell-derived vesicles from the circulation by scavenger receptors. Blood..

[27] Xing Li, Cui Rui, Peng Lei, Ma Jing, Chen Xiao, Xie Ru-Juan, Li Bing (2014). Mesenchymal stem cells, not conditioned medium, contribute to kidney repair after ischemia-reperfusion injury. Stem Cell Research & Therapy.

[28] Hualin M, Shuyan Z, Ying X, Rongrong Z, Xinzhou Z (2018). Analysis of differentially expressed microRNA of TNF-α-stimulated mesenchymal stem cells and exosomes from their culture supernatant. Arch Med Sci..

[29] Fernandez-Pernas. P (2017). CD105+-mesenchymal stem cells migrate into osteoarthritis joint: An animal model. PLoS One..

[30] Mahrouf-Yorgov. M (2017). Mesenchymal stem cells sense mitochondria released from damaged cells as danger signals to activate their rescue properties. Cell Death Differ..

[31] Agung M (2006). Mobilization of bone marrow-derived mesenchymal stem cells into the injured tissues after intraarticular injection and their contribution to tissue regeneration. Knee Surg Sports Traumatol Arthrosc..

[32] Eom. YW (2014). The role of growth factors in maintenance of stemness in bone marrow-derived mesenchymal stem cells. Biochem Biophys Res Commun..

[33] Wang. L, Lazebnik M, Detamore MS (2009). Hyaline cartilage cells outperform mandibular condylar cartilage cells in a TMJ fibrocartilage tissue engineering application. Osteoarthritis Cartilage..

[34] Blackstock. CD (2014). Insulin-like growth factor-1 increases synthesis of collagen type I via induction of the mRNA-binding protein LARP6 expression and binding to the 5′ stem-loop of COL1a1 and COL1a2 mRNA. J Biol Chem..

[35] Ozeki N (2016). Not single but periodic injections of synovial mesenchymal stem cells maintain viable cells in knees and inhibit osteoarthritis progression in rats. Osteoarthritis Cartilage..

[36] Li M (2016). *In vivo* human adipose-derived mesenchymal stem cell tracking after intra-articular delivery in a rat osteoarthritis model. Stem Cell Res Ther..

[37] Zwolanek. D (2017). Tracking mesenchymal stem cell contributions to regeneration in an immunocompetent cartilage regeneration model. JCI Insight..

[38] Paliwal. S, Chaudhuri. R, Agrawal. A, Mohanty. S (2018). Regenerative abilities of mesenchymal stem cells through mitochondria transfer. J Biomed Sci..

[39] Spees. JL, Olson SD, Whitney MJ, Prockokp. DJ (2006). Mitochondrial transfer between cells can rescue aerobic respiration. Proc Natl Acad Sci USA.

[40] Blanco. FJ, Rego I, Ruiz-Romero C (2011). The role of mitochondria in osteoarthritis. Nat Rev Rheumatol..

[41] Delco. ML, Bonnevie ED, Bonassar LJ, Fortier LA (2018). Mitochondrial dysfunction is an acute response of articular chondrocytes to mechanical injury. J Orthop Res..

[42] Hare. JM (2012). Comparison of allogeneic vs autologous bone marrow-derived mesenchymal stem cells delivered by transendocardial injection in patients with ischemic cardiomyopathy: the poseidon randomized trial. JAMA..

[43] Park. EH (2018). Intravenous infusion of umbilical cord blood – derived mesenchymal stem cells in rheumatoid arthritis: a phase Ia clinical trial. Stem Cells Transl Med..

[44] Güler N, Kürkçü M, Duygu G, Cam B (2011). Sodium iodoacetate induced osteoarthrosis model in rabbit temporomandibular joint: CT and histological study (Part I). Int J Oral Maxillofac Surg..

[45] Cui Dixin, Li Hongyu, Xu Xin, Ye Ling, Zhou Xuedong, Zheng Liwei, Zhou Yachuan (2017). Mesenchymal Stem Cells for Cartilage Regeneration of TMJ Osteoarthritis. Stem Cells International.

[46] Guzman RE, Evans MG, Bove S, Morenko B, Kilgore K (2003). Mono-iodoacetate induced histologic changes in subchondral bone and articular cartilage of rat femorotibial joints: an animal model of osteoarthritis. Toxicol Pathol..

[47] Kim J (2015). Umbilical cord mesenchymal stromal cells affected by gestational diabetes mellitus display premature aging and mitochondrial dysfunction. Stem Cells Dev..

[48] Park BW (2012). *In vitro* and *in vivo* osteogenesis of human mesenchymal stem cells derived from skin, bone marrow and dental follicle issues. Differentiation..

