# Supplementary material for: Therapeutic effect of mesenchymal stem cells derived from human umbilical cord in rabbit temporomandibular joint model of osteoarthritis
Source: Sci Rep. 2019 Sep 25;9:13854. doi: 10.1038/s41598-019-50435-2 (PMC6761110; doi:10.1038/s41598-019-50435-2)
Supplement: Supplementary file 1 — Supplementary information data [file 41598_2019_50435_MOESM1_ESM.pdf]

Therapeutic effect of mesenchymal stem cells derived from the human umbilical cord in a rabbit temporomandibular joint model of osteoarthritis

Hyunjeong Kim[1], Gwanghyun Yang[1], Jumi Park[2], Jene Choi[3], Eunju Kang[4], Bu-Kyu Lee[1, 4, 5]*

[1]Biomedical Engineering Research Center, Asan Institute for Life Sciences, Asan Medical Center, College of Medicine, University of Ulsan, Seoul, Republic of Korea

[2]Department of Stem Cell Center, Asan Institute for Life Sciences, Asan Medical Center, Seoul, Republic of Korea

[3]Department of Pathology, Asan Medical Center, College of Medicine, University of Ulsan, Seoul, Republic of Korea

[4]Department of Stem Cell Center, Asan Institute for Life Science, Asan Medical Center, College of Medicine, University of Ulsan, Seoul, Republic of Korea

[5]Department of Oral and Maxillofacial Surgery, Asan Medical Center, College of Medicine, University of Ulsan, Seoul, Republic of Korea

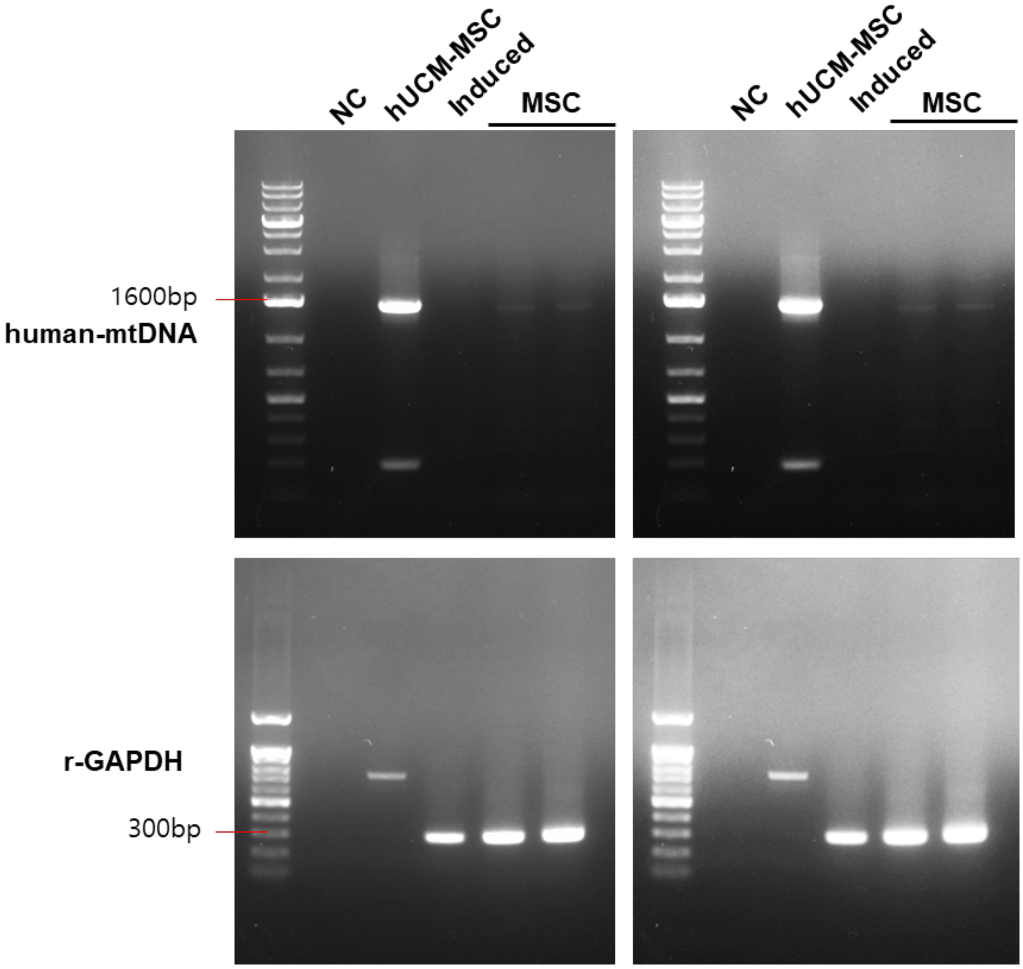
NC
hUCM-MSC
Induced
MSC
NC
hUCM-MSC
Induced
MSC
1600bp
human-mtDNA
r-GAPDH
300bp
